# *ERBB2* mutations associated with solid variant of high-grade invasive lobular breast carcinomas

**DOI:** 10.18632/oncotarget.11819

**Published:** 2016-09-02

**Authors:** Gabrielle Deniziaut, Jean Christophe Tille, François-Clément Bidard, Sophie Vacher, Anne Schnitzler, Walid Chemlali, Laurence Trémoulet, Laetitia Fuhrmann, Paul Cottu, Roman Rouzier, Ivan Bièche, Anne Vincent-Salomon

**Affiliations:** ^1^ Institut Curie, PSL Research University, Pathology-Genetics-Immunology Department, 75005 Paris, France; ^2^ Geneva University Hospital, Faculty of Medicine, Division of Clinical Pathology, 1205 Geneva, Switzerland; ^3^ Institut Curie, PSL Research University, Medical Oncology Department, 75005 Paris, France; ^4^ Institut Curie, Versailles Saint-Quentin University, Surgery Department, 92210 Saint-Cloud, France; ^5^ Institut Curie, PSL Research University, INSERM U934, 75005 Paris, France

**Keywords:** *ERBB2* mutations, *ERBB3* mutations, invasive lobular breast carcinoma, sequencing, anti-ERBB2 targeted agents

## Abstract

*ERBB2* and *ERBB3* somatic gain-of-function mutations, which may be targeted by anti-ERBB2 therapies, were reported by high-throughput sequencing studies in 1% and 2% of invasive breast cancers respectively. Our study aims to determine *ERBB2* and *ERBB3* mutations frequencies in grade 3 and/or ERBB2-positive invasive lobular breast carcinomas (ILC). All the 529 ILC surgically-excised registered at Institut Curie in the years 2005 to 2008 were reviewed. Thirty-nine grade 3 ERBB2-negative ILC and 16 ERBB2-positive ILC were retrieved and subjected to Sanger sequencing of the *ERBB2* and *ERBB3* activation mutation hotspots (*ERBB2*: exons 8, 17, 19, 20, 21; *ERBB3*: exons 3, 6, 7, 8). Among the 39 grade 3 ERBB2-negative ILC, six tumors were found to have at least one detectable *ERBB2* activating mutation (incidence rate: 15%, 95%CI [4%-27%]). No *ERBB2* mutation was found among the 16 ERBB2-positive ILC. No *ERBB3* mutation was found in any of the 55 ILC. *ERBB2* mutations were statistically associated with solid ILC features (p=0.01). Survival analyses showed no significant prognostic impact of *ERBB2* mutations. Our study demonstrates that high grade ERBB2-negative ILC display a high frequency of *ERBB2* mutations, and should be subjected to systematic genetic screening.

## INTRODUCTION

The human epidermal growth factor receptor (HER) family is composed of four transmembrane receptors: EGFR/HER1, ERBB2/HER2, ERBB3/HER3 and ERBB4/HER4 [1]. Schematically, these proteins have an extra-cellular domain that can bind different growth factors, a single hydrophobic transmembrane segment (α-helix), and an intracellular portion that includes a protein kinase domain [2, 3]. The intracellular kinase activity is activated by the binding of extracellular ligands and/or homo- or hetero dimerization of the receptor with another member of the family [3]. When activated, these receptors stimulate multiple downstream signaling pathways, such as those involving mitogen-activated protein kinase and phosphatidylinositol-3 kinase, and finally lead to cell growth and survival [4]. However, ERBB2 has no known ligand and its intracellular kinase activity is exclusively activated by homo- or heterodimerization [5]. In contrast, ERBB3 displays no significant intracellular kinase activity and signals through heterodimerization, mainly with ERBB2 [6, 7].

The key oncogenic role of *ERBB2* amplification in a subset of breast cancer has been described in the late 80′s [8]. *ERBB2*-amplified breast cancers have been since isolated as a molecular subgroup representing about 15% of invasive breast cancers, usually of high tumor grade and exhibiting a poor survival [9]. In the early 2000′s, the ERBB2 targeting monoclonal antibody trastuzumab demonstrated significant improvements of survival in *ERBB2*-amplified breast cancer patients, leading to a “revolution” in medical oncology [10]. Clinical studies with other ERBB2-targeting drugs, either antibodies (e.g. pertuzumab, T-DM1) or tyrosine kinase inhibitors (e.g. lapatinib, neratinib…), later demonstrated that metastatic *ERBB2*-amplified breast cancer cells display an “oncogenic addiction” and that anti-ERBB2 therapy should be pursued despite disease progression. These major clinical advances were made possible by the standardization of breast cancer ERBB2-status testing by pathologists, based either on direct *ERBB2* copy number assessment (by *in-situ* hybridization techniques) or on its validated surrogate, ERBB2 protein overexpression (by standardized immunohistostaining) [11].

With the advent of massively parallel sequencing, mutations in the sequence of the *ERBB2* gene were found in a limited fraction (<1%) of breast cancers [12]. *In vitro* functional characterization of these mutations demonstrated that some of them had oncogenic properties and promoted cancer cells growth, invasion and survival [13–15]. Moreover, *ERBB2* activating mutations conferred *in vitro* sensibility to anti-ERBB2 drugs, particularly but not exclusively to the second generation tyrosine kinase inhibitor neratinib, even in the absence of any *ERBB2* amplification [13, 14]. Following this seminal report, several other *ERBB2* mutations were reported, together with their *in vitro* or in patient sensitivity to ERBB2-targeted therapy [16, 17]. Similarly, activating point mutations of *ERBB3* have been described in 2013 and were found *in vitro* to display oncogenic activities in breast cancer cells [18]. ERBB3 being unable to initiate an intracellular signal on its own, Jaiswal and colleagues demonstrated that ERBB3 mutants signal through heterodimerization with ERBB2, and that anti-ERBB2 therapies efficiently blocked ERBB3-initiated oncogenic signaling in cell lines. Our group reported a few months ago the first case of a patient who was treated by trastuzumab and lapatinib as third line regimen for ERBB2-negative metastatic breast cancer on the basis of an activating *ERBB3* mutation retrieved by all-exome sequencing in both the primary tumor and liver metastases [19]. After only two weeks of dual ERBB2 blockade, the patient exhibited a complete metabolic response followed by a 20 months progression-free interval, although no chemotherapy was used.

In light of the above-mentioned preclinical and clinical results, *ERBB2* and *ERBB3* activating mutations are now considered as “targetable” by anti-ERBB2 drugs, but further clinical evidences are needed. Most of industry- and academia-developed targeted sequencing panels now include these 2 genes, and one international trial (NCT01953926) has been set up to study the efficacy of neratinib on these mutations (among other mutations of the ERBBs family). The main limitation of such approach is the rarity of *ERBB2* and *ERBB3* activating mutations among breast cancers. Previous reports suggested that *ERBB2* mutations might be enriched in a specific histological subtype, invasive lobular carcinoma (ILC) [12, 20], which account for 5-15 % of invasive breast tumors [21, 22]. ILC are characterized by a proliferation of non-cohesive cancer cells and usually display low tumor grade, high hormone receptors expression and a lower rate of *ERBB2* amplification than invasive breast carcinomas of non-specific type [23]. We therefore hypothesized that *ERBB2* and *ERBB3* activating mutations might be over-represented in ILC harboring atypical features such as high tumor grade or *ERBB2* amplification. The aim of our study was to report the detection rate of *ERBB2* and *ERBB3* mutations in a consecutive series of high grade or ERBB2-positive ILC.

## RESULTS

**Figure 1:**
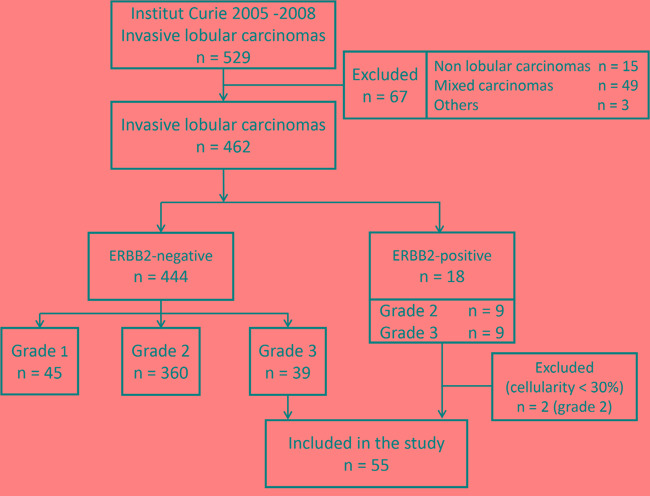
Study flow chart

The sample flow chart is shown in Figure 1. We retrospectively retrieved 529 tumors diagnosed as a primary breast ILC excised from women treated at the Institut Curie (France) from 2005 to 2008. After histological review, 67 (13%) tumors were excluded (15 non ILC, 49 carcinomas with a ductal component above 10%, three multifocal or multicentric carcinomas with non-lobular invasive component). After immunohistochemical analyses (eventually completed by FISH), 18 of the remaining 462 ILC (4%) were ERBB2-positive (nine tumors of grade 2, nine of grade 3) in agreement with the literature. Two grade 2 ERBB2-positive samples were excluded because of a cellularity below 30%. Among the 444 ERBB2-negative ILC, 45 were of grade 1 (10%), 360 of grade 2 (81%) and 39 of grade 3 (9%). We therefore included a total of 55 ILC in our study, including 16 ERBB2-positive ILC and 39 grade 3 ERBB2-negative ILC. The corresponding clinicopathological characteristics are shown in Table 1; 51 tumors (93%) were estrogen receptors (ER)-positive, 40 tumors (73%) were progesterone receptors (PR)-positive and 53 tumors (96%) were E-cadherin-negative, while the median age at time of diagnosis was 62 years (range: 37-87 years).

**Table 1:** Clinicopathological characteristics and *ERBB2* mutations

Characteristics	All patients Number (%)	*ERBB2*_Wild type_ Number (%)	*ERBB2*_mutated_ Number (%)	p-value^(1)^
Total	55 (100)	49 (89)	6 (11)	
Median Age (range)	62 (37 - 87)	62 (37 - 87)	61 (58 - 84)	
Type				
Mixed	26 (47)	24 (92)	2 (8)	0,01
Classic	22 (40)	21(95)	1 (5)	
Solid	6 (11)	3 (50)	3 (50)	
Pleomorphic	1 (2)	1 (100)	0 (0)	
Apocrine differentiation				
No	45 (82)	40 (89)	5 (11)	1
Yes	10 (18)	9 (90)	1 (10)	
Grade				
2	7 (13)	7 (100)	0 (0)	1
3	48 (87)	42 (87)	6 (13)	
Mitotic index				
1	7 (13)	7 (100)	0 (0)	0,4
2	27 (49)	25 (96)	2 (7)	
3	21 (38)	17 (81)	4 (19)	
LVI^(2)^				
No	39 (71)	34 (87)	5 (13)	0,7
Yes	16 (29)	15 (94)	1 (6)	
pT				
pT1	25 (46)	23 (92)	2 (8)	0,8
pT2	20 (36)	17 (85)	3 (15)	
pT3	10 (18)	9 (90)	1 (10)	
pN				
pN0	33 (60)	29 (88)	4 (12)	0,2
pN1	9 (16)	7 (78)	2 (22)	
pN2-N3	13 (234)	13 (100)	0 (0)	
ER				
Negative	4 (7)	3 (75)	1 (25)	0,4
Positive	51 (93)	46 (90)	5 (10)	
PR				
Negative	15 (27)	13 (87)	2 (13)	0,7
Positive	40 (73)	36 (90)	4 (10)	
*ERBB2*				
Non amplified	39 (71)	33 (85)	6 (15)	0,2
Amplified	16 (29)	16 (100)	0 (0)	
E-cadherin				
Negative	53 (96)	47 (89)	6 (11)	1
Positive	2 (4)	2 (100)	0 (0)	

(1) Fisher's exact test

(2) LVI : Lympho-vascular invasion

**Figure 2:**
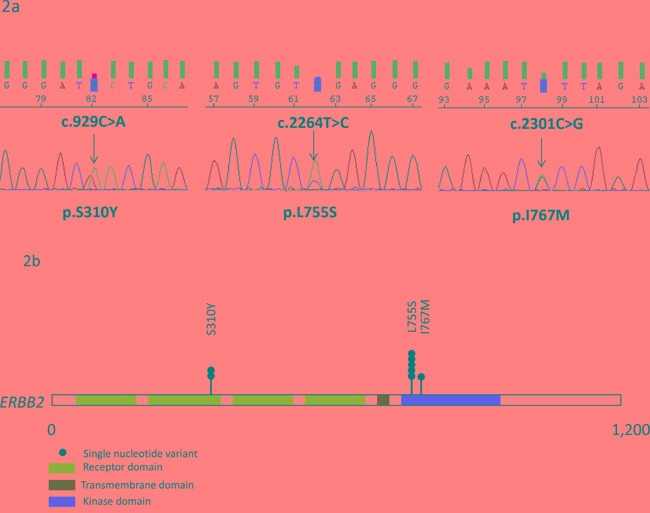
Mutations identified in a series of 55 invasive lobular carcinomas **a.** Electropherograms of 3 cases with *ERBB2* mutation. **b.** Distribution of *ERBB2* mutations

Among the 55 ILC subjected to sequencing, six (11%, 95%CI[3; 19]) were found to have at least one *ERBB2* missense somatic mutation. Notably, these mutations were observed only in grade 3 ERBB2-negative ILC (6/39; 15.4%, 95%CI[4;27]). Four tumors displayed the p.L755S (c.2264T>C) missense mutation, whereas two tumors had two mutations, p.L755S and p.S310Y (c.929C>A) for one and p.I767M (c.2301C>G) and p.S310Y for the other (Figure 2a). Reverse sequencing also confirmed these results. Thus, we identified six mutations in the kinase domain (p.L755S (five out of eight mutations; 63%) and p.I767M) and two mutations in the extracellular domain of the receptor (p.S310Y) (Figure 2b).

In stark contrast to these findings, no *ERBB3* activating mutation was found in any of the 55 tumor samples. The validity of the *ERBB3* sequencing method was successfully checked by sequencing the *ERBB3*-mutated breast cancer case (G284R) mentioned in the introduction [19] and used as a positive control.

We then investigated the association of *ERBB2* mutation status and the clinicopathological variables (Table 1). The solid subtype of ILC was significantly associated with *ERBB2* mutation (Fisher's exact test; *p = 0.01*). Solid subtype was observed in 50% (3/6) of mutant *ERBB2* tumors versus 6% (3/49) of wild type *ERBB2* tumors (Fisher's exact test; *p = 0.01*). One out of the 10 ILC with apocrine differentiation was mutated. No significant correlation was found between the mutant *ERBB2* status and other classical clinicopathological parameters such as age, Elston-Ellis tumor grade, mitotic index, presence of lymphovascular invasion, macroscopic tumor size, lymph node status, ER, PR and E-cadherin expression. Four mutated ILC expressed ER and PR, one expressed only ER and the last one was triple negative. All mutated ILC were E-cadherin-negative by immunohistochemistry. We observed that all *ERBB2* mutations were found in ERBB2-negative samples, although this possible mutual exclusion did not reach statistical significance (*Fisher's exact test p = 0.2*). We therefore report a 15.4% (6/39, 95%CI[4%; 27%]) *ERBB2* mutations rate in grade 3 ERBB2-negative ILC versus 0% (0/16) in grade 3 ERBB2-positive ILC.

**Figure 3:**
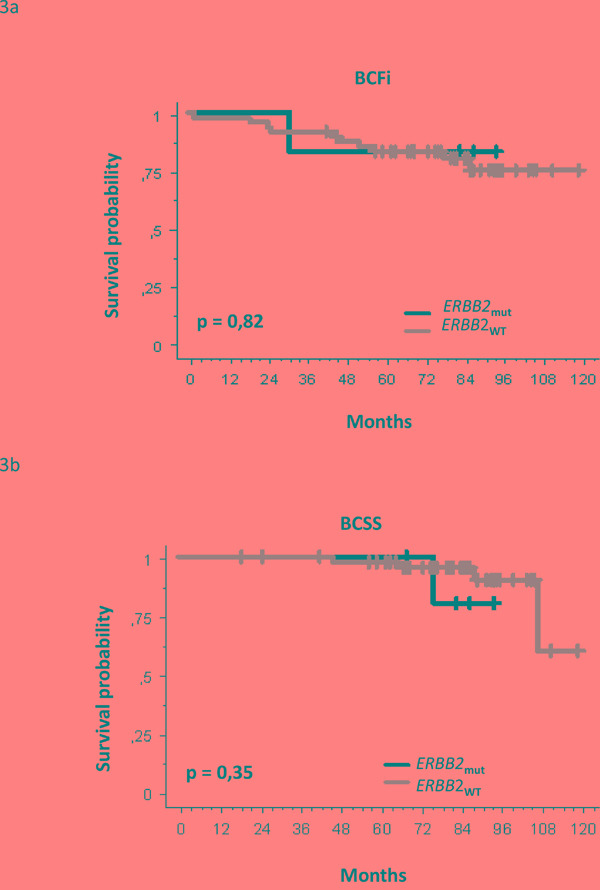
Survival analysis in the mutated and wild-type ERBB2 patients subgroups **a.** Breast cancer free interval (BCFi) analysis. **b.** Breast cancer specific survival (BCSS) analysis

We finally studied the effect of *ERBB2* mutational status on long-term outcome. With a median follow-up of 84 months (range: 19 – 121 months), 5 deaths (9%) and 11 tumor progressions (20%) have been recorded at time of analysis (October 2015). This exploratory analysis showed no significant prognostic impact of *ERBB2* mutation on breast cancer-free interval and breast cancer specific survival (*p = 0.82* and *0.35* respectively, Figure 3). Similar results were observed when including only the 39 ERBB2-negative ILC in the survival analysis.

## DISCUSSION

Based on a large consecutive series, our study demonstrates that activating *ERBB2* mutations are particularly enriched (15.4% detection rate, 95%CI[4;27]) in grade 3 ERBB2-negative ILC. This mutation rate is far above the ≤1% *ERBB2* mutation rate globally reported for breast cancer in The Cancer Genome Atlas [12]. Interestingly, a similar rate of *ERBB2* mutations was previously reported in a smaller series by Lien and colleagues, who identified *ERBB2* mutations in five out of 24 (21%) invasive and *in-situ* pleomorphic lobular carcinomas [24].

Our study focused on lobular carcinomas predominantly E-cadherin-negative with only two E-cadherin-positive ILC cases. This composition of our series did not allow identifying any significant correlation between *ERBB2* mutations and E-cadherin loss of expression by immunohistochemistry. Interestingly enough, using the cBioportal, we found that the 9 *ERBB2* mutated ILC cases of TCGA 2015 were *CDH1* mutated with a significant correlation with a Fisher's exact test (*p = 0.02*) calculated among breast carcinomas of other histological subtypes.

In our study, the solid subtype of ILC appears as frequently mutated, as half of the six solid ILC had at least one *ERBB2* mutation. This link between an ILC subtype and more frequent *ERBB2* mutations has been also suggested by Desmedt et al [25]. However, the first TCGA study identified *ERBB2* mutations in grade 2 and 3 classic ILC (Supplementary Table 1).

Additional series are needed to confirm the preferential association on the one hand between *ERBB2* mutations and solid subtype of ILC and on the other hand between *ERBB2* mutations and *CDH1* mutations.

In contrast to Lien et al, apocrine differentiation was not associated with *ERBB2* mutations. Also, our result suggests that *ERBB2* mutation and amplification are two mutually exclusive oncogenic events in ILC. However, a few breast cancers with coexisting *ERBB2* mutation and homogeneous or heterogeneous amplification have been already reported [12, 26].

Regarding mutation distribution, the p.L755S mutation represented five of the eight (63%) *ERBB2* mutations identified in this study. The L755 residue has been previously reported by Bose et al as the most frequent mutation hotspot in the *ERBB2* gene [14]. Although the oncogenic activity of the L755 mutations was not demonstrated, Bose et al reported that mutated L755 breast cancer cell lines were less sensitive to the reversible dual *EGFR/ERBB2* inhibitor lapatinib but may be sensitive to irreversible dual *EGFR/ERBB2* inhibitors such as neratinib [14, 15]. Regarding the other mutations found in our series, the p.I767M mutation was also not functionally characterized as oncogenic, but appeared sensitive to conventional anti-ERBB2 drugs (trastuzumab, lapatinib, neratinib) [14]; the p.S310Y mutation has been functionally characterized as activating mutations that sensitize cancer cells to ERBB2 inhibitors [13]. Interestingly, the mutational spectrum of *ERBB2* of ILC reported in this study is distinct from that reported previously by studies carried out on Asian populations, suggesting a possible influence of ethnic factors on the *ERBB2* mutation spectrum [24, 27, 28]. Notably, a case-report described the spectacular response to lapatinib for a metastatic ILC patient harboring a rare *ERBB2* mutation (p.L869Q) [17]. Altogether these results demonstrate the interest of *ERBB2* mutations screening in clinical practice.

A comprehensive analysis of ILC has been recently published by Ciriello et al [29]. Their study included 127 ILC of any grade and reported five *ERBB2* mutations (4% mutation rate). Our study originates from a collection of 462 reviewed ILC of any grade and focused on the small grade 3 ERBB2-negative subgroup, which account for less than 10% of ILC. It might therefore be possible that the large and very exhaustive analysis published by Ciriello et al had not enough power to detect the association between *ERBB2* mutations and high grade ILC.

More recently, Michaut et al performed comprehensive molecular profiling of 144 primary ILC tumors and described two biologically distinct subtypes of ILC: the Immune Related subtype defined by up-regulation of genes implicated in cytokine/chemokine signaling and the Hormone Related subtype, characterized by higher levels of estrogen and progesterone receptors and up-regulation of cell cycle genes and estrogen receptor target genes. *ERBB2* mutations were found in 4% of ILC, in line with a number of previous studies. They were associated with the Hormone Related subtype [30].

In contrast to Desmedt et al. that reported a 3.6% (15/413) mutation of *ERBB3* in ILC [25], we did not find any *ERBB3* activating mutation among the 55 tested ILC. Our study was not designed to detect the E928G mutation reported by Desmedt. Our results suggest that *ERBB3* mutations are not enriched in grade 3 ILC, and may rather be found in ILC of lower grade. Notably, Desmedt et al. did not find a link between *ERBB3* mutations and high grade ILC. ICGC data showed that, among breast cancers, *ERBB3* activating mutations are predominantly found in ILC and / or triple negative breast carcinomas but remain a rare event in breast carcinomas [31].

A last interesting finding is that, although of limited size, our cohort had a long clinical follow-up after surgery; this allowed performing an exploratory survival analysis which showed no major prognostic impact of *ERBB2* mutations.

To conclude, our study demonstrates that, in the era of personalized therapy, rare targetable activating mutations can be significantly enriched in specific breast tumor subtypes. With a very high *ERBB2* mutation rate of 15.4%, patients with ERBB2-negative ILC should be screened and grade 3 addressed to personalized therapeutic trials whenever possible.

## MATERIALS AND METHODS

### Patients and samples

This study was approved by the local ethics committee (Breast Group of Institut Curie); no informed consent was required because of the retrospective nature of the study. Using the electronic database of Institut Curie, we selected all cases diagnosed as primary breast ILC which were surgically operated between January 2005 and December 2008. Tumor biopsies and primary tumors previously exposed to neoadjuvant therapy were excluded. All slides were reviewed by two experienced breast pathologists (AVS and JCT) and cases were classified meticulously into classic, solid, pleomorphic or mixed lobular carcinomas according to the World Health Organization criteria [32]. When present, apocrine morphologic features were described. Non-lobular carcinomas, mixed carcinomas with a ductal component above 10%, multifocal and multicentric carcinomas with non-lobular components were further excluded after pathological review. ILC tumor grade was determined following the Elston-Ellis grading system [33]. Only grade 3 and ERBB2-positive ILC were finally included in this study.

### Immunohistochemistry staining

All cases included in this study were reviewed for ER and PR, ERBB2 and E-cadherin expression. Diagnostic immunostainings were reviewed and further immunostainings were performed when missing, using the routine diagnostic procedure. In brief, immunostaining was performed on 4 μm tissue sections prepared from a representative sample of the tumor. After rehydration and antigenic retrieval in citrate buffer (10 mM, pH 6.1), tissue sections were stained for E-cadherin (E-cadh, clone HECD1, Zymed Laboratories Inc., 1/50) when necessary to confirm ILC diagnosis, according to the WHO classification (24), ER (clone 6F11, Novocastra, 1/200), PR (clone 1A6, Novocastra, 1/200) and ERBB2 (CB11, Leica Biosystems, 1/100). Revelation of staining was performed using the Vectastain Elite ABC peroxidase mouse IgG kit (Vector Burlingame, CA) and diaminobenzidine (Dako A/S, Glostrup, Denmark) as chromogen. Positive and negative controls were included in each slide run. Cases were considered negative for E-cadherin when no tumor cell presented any strong membrane staining in the presence of accurate external/internal positive controls (surrounding normal glands). Cases were considered positive for ER and PR according to standardized guidelines using a percentage of 10% of positive nuclei [34–36]. A tumor was considered ERBB2-positive by immunohistochemistry if it scored 3 with uniform intense membrane staining of greater than 10% of invasive tumor cells. Tumors scoring 2 were considered to be equivocal for ERBB2 protein expression and were tested by fluorescence *in situ* hybridization for *ERBB2* gene amplification [37].

### DNA extraction

Prior to DNA isolation, the tumor cellularity was evaluated on hemalun-eosin-safran stained sections. Tumor samples were considered suitable for this sequencing study only if they showed >30% of tumor cells after manual macrodissection. For each case, one representative area with adequate tumor cellularity was marked out on the slide. Using a tissue arrayer, 3 mm diameters cores of breast cancer tissue was punched out from the Formalin Fixed and Paraffin Embbeded (FFPE) tissue block. DNA was extracted from FFPE samples using the NucleoSpin^®^ 8 tissue kit (Macherey-Nagel, Germany). DNA quantity was assessed with the Qubit dsDNA HS Assay kit (Life technologies). DNA quality was controlled by qPCR.

### *ERBB2* and *ERBB3* sequencing

*ERBB2* and *ERBB3* mutations were detected in each tumor by screening DNA fragments obtained by PCR amplification of exons 8, 17, 19, 20 and 21 for *ERBB2* and 3, 6, 7 and 8 for *ERBB3* using classical Sanger method. The primer sequences are available in Supplementary Table 2. They were designed to allow identification of most prevalent *ERBB2* and *ERBB3* mutations, including S310F/Y, R678Q, L755M/P/S/W, V777A/L/M and V842I mutations in *ERBB2* and M91I, V104M/L, N126K, H228Q/R, A232V/T, G284R/G, D297N/V/Y mutations in *ERBB3*. Each PCR was performed on 1, 2 μL of tumor DNA. Purified PCR products were bidirectionally sequenced with dideoxynucleotides using BigDye Terminator V1.1 chemistry (Applied Biosystems) and 20 μM of specific primer in a 10, 2 μL total volume, on a Gene Amp^®^ PCR system 9700 (Applied Biosystems), purified on a Sephadex G50 column, and analyzed with a capillary sequencing machine (3500 XL Genetic Analyzer, Applied Biosystems). Sequences were then examined with Sequencing analysis software (Applied Biosystems) and compared to the corresponding DNA reference sequence (NM_004448.3 for *ERBB2* and NM_001982.3 for *ERBB3*). Mutations were detected with a sensitivity of 10% mutated alleles. All of the detected mutations were confirmed in a second independent run of sample testing.

### Statistical analyses

Patient characteristics and outcomes were retrieved from the electronic medical file for all patients. Differences between categorical variables were analyzed by a Fisher's exact test. Breast Cancer Free interval (BCFi) was determined as the interval from diagnosis of breast cancer to any breast cancer event including local, regional or distant recurrence or contralateral disease. Breast Cancer Specific Survival (BCSS) was defined as the time from diagnosis of breast cancer to death due to breast cancer. Survival curves were plotted according to the Kaplan–Meier method. Statistical significance between survival curves was assessed using the logrank test. For all analyses, a p value of ≤0.05 was considered to be statistically significant. Regarding the sample size, no attempt was made in this study to define target statistical power.

## SUPPLEMENTARY MATREIALS TABLES


