# Supplementary material for: ERBB2 mutations associated with solid variant of high-grade invasive lobular breast carcinomas
Source: Oncotarget. 2016 Sep 2;7(45):73337–46. doi: 10.18632/oncotarget.11819 (PMC5341983; doi:10.18632/oncotarget.11819)
Supplement: Supplementary file 1 [file oncotarget-07-73337-s001.pdf]

ERBB2 mutations associated with solid variant of high-grade invasive lobular breast carcinomas

SUPPLEMENTARY TABLES

Supplementary Table S1: We analysed the 9 cases with ERBB2 mutation from the TCGA 2015 study using the Cancer Digital Slide archive (cancer.digitalslidearchive.net/) recovered from the cBioportal (http://www.cbioportal.org/). Grade and morphologic subtype were done and it is summarized in the following table:

Case ID	*ERBB2* mutation	Grade	Differenciation	Nucleus	Mitosis	Subtype
TCGA-OK-A5Q2-01	L755S	G2	3	2	1	Mixed classic alveolar
TCGA-JL-A3YW-01	Q548R	G2	3	3	1	Solid
TCGA-D8-A27G-01	I767M	G2	3	3	1	Classic apocrine
TCGA-C8-A3M7-01	S305C	G2	3	2	1	Classic
TCGA-BH-A0C1-01	V777L	G2	3	3	1	Classic
TCGA-AC-A3YI-01	L755S	G2	3	2	1	Classic
TCGA-A8-A0AB-01	L755S	G3	3	3	3	Mixed classic trabecular
TCGA-A8-A0A6-01	L755S	Frozen section of too poor quality to be correctly analyzed				
TCGA-A2-A0T6-01	R678Q, L755R	G2	3	2	1	Mixed classic solid

Few preliminary remarks can be done:

- ⇨ The 9 *ERBB2* mutated cases are split as follows: 7 were of grade 2, 1 of grade 3 and 1 was not analyzed.
- ⇨ In addition, out of these 9 *ERBB2* mutated cases, 4 were of classical subtype, 3 were of mixed subtype, 1 was of solid subtype and 1 was not analyzed.

The population is too small to draw any meaningful conclusion at this stage. However, it reinforces the idea that we should analyze the whole cohort including grade 1 and 2 ILC in a later study.

Supplementary Table S2: Primer sequences

Gene	Detectable mutations	Primer sequences U(5'-3') and L(5'-3')	Amplicon size (nt)
D-*ERBB2* Exon 8	p.S310F c.929 C>T; p.S310Y c.929 C>A	U : GGGCACGGTAATGCTGCTCA<br>L : CAGGGCTTGCTGCACTTCTCA	185
D-*ERBB2* Exon 17	p.R678Q c.2033G>A	U : GCATTCTGCTGGTCGTGGTCTT<br>L : GACTTCACCCCGCCTCACCT	125
D-*ERBB2* Exon 19	p.L755M c.2263 T>A; p.L755P c.2263_2264 TT>CC; p.L755S c.2264 T>C; p.L755W c.2264 T>G	U : TTCTCACTCATATCCTCCTCTTTCTG<br>L : GGGGTCCTTCCTGTCCTCCT	175
D-*ERBB2* Exon 20	p.V777A c.2330 T>C; p.V777L c.2329 G>T; p.V777M c.2329 G>A	U : TGTGGTCTCCCATACCCTCTCAG<br>L : TGTCAGGCAGATGCCCAGAAG	106
D-*ERBB2* Exon 21	p.V842I c.2524 G>A	U : CAGGCCCTCCCAGAAGGTCTA<br>L : AGCCCGAAGTCTGTAATTTTGACAT	146
D-*ERBB3* Exon 3	p.M91I c.273 G>A; p.V104M c.310 G>A; p.V104L c.310 G>T; p.N126I c.377 A>T; p.N126K c.378 C>T	U : TCACAGTGGATTCGAGAAGTGACA<br>L : ACTGACCGGTGAGCTGAGTCAA	197
D-*ERBB3* Exon 6	p.H228Q c.684 T>A; p.H228R; p.A232V c.695 C>T; p.A232T c694G>A	U : CTGTGCTCCTCAGTGTAATGGTCA<br>L : CAATGGAAAGGGTACATACAAAGCA	125
D-*ERBB3* Exon 7	p.G284R c.850 G>A; p.G284G c.852 A>G	U : GAACCCAATCCCCACACCAAGT<br>L : TGATCATTGTTCCTTCCCCTCAGA	89
D-*ERBB3* Exon 8	p.D297N c.889 G>A; p.D297Y c.889 G>T; p.D297V c.890 A>T	U : CATTGGTAGCTGGTGATGTTCCT<br>L : TCCCCCACAAGGCTCACACAT	161
